# Development of a Semi-automated Computer-based Tool for the Quantification of Vascular Tortuosity in the Murine Retina

**DOI:** 10.1016/j.xops.2023.100439

**Published:** 2023-12-06

**Authors:** Kyle V. Marra, Jimmy S. Chen, Hailey K. Robles-Holmes, Joseph Miller, Guoqin Wei, Edith Aguilar, Yoichiro Ideguchi, Kristine B. Ly, Sofia Prenner, Deniz Erdogmus, Napoleone Ferrara, J. Peter Campbell, Martin Friedlander, Eric Nudleman

**Affiliations:** 1 Department of Molecular Medicine, The Scripps Research Institute, San Diego, California; 2 School of Medicine, University of California San Diego, San Diego, California; 3 Department of Ophthalmology, Shiley Eye Institute, University of California San Diego, San Diego, California; 4 College of Optometry, Pacific University, Forest Grove, Oregon; 5 Department of Electrical and Computer Engineering, Northeastern University, Boston, Massachusetts; 6 Department of Ophthalmology, Casey Eye Institute, Oregon Health & Science University, Portland, Oregon

**Keywords:** Vessel tortuosity, Oxygen-induced retinopathy, Computer-based image analysis

## Abstract

**Purpose:**

The murine oxygen-induced retinopathy (OIR) model is one of the most widely used animal models of ischemic retinopathy, mimicking hallmark pathophysiology of initial vaso-obliteration (VO) resulting in ischemia that drives neovascularization (NV). In addition to NV and VO, human ischemic retinopathies, including retinopathy of prematurity (ROP), are characterized by increased vascular tortuosity. Vascular tortuosity is an indicator of disease severity, need to treat, and treatment response in ROP. Current literature investigating novel therapeutics in the OIR model often report their effects on NV and VO, and measurements of vascular tortuosity are less commonly performed. No standardized quantification of vascular tortuosity exists to date despite this metric’s relevance to human disease. This proof-of-concept study aimed to apply a previously published semi-automated computer-based image analysis approach (iROP-Assist) to develop a new tool to quantify vascular tortuosity in mouse models.

**Design:**

Experimental study.

**Subjects:**

C57BL/6J mice subjected to the OIR model.

**Methods:**

In a pilot study, vasculature was manually segmented on flat-mount images of OIR and normoxic (NOX) mice retinas and segmentations were analyzed with iROP-Assist to quantify vascular tortuosity metrics. In a large cohort of age-matched (postnatal day 12 [P12], P17, P25) NOX and OIR mice retinas, NV, VO, and vascular tortuosity were quantified and compared. In a third experiment, vascular tortuosity in OIR mice retinas was quantified on P17 following intravitreal injection with anti-VEGF (aflibercept) or Immunoglobulin G isotype control on P12.

**Main Outcome Measures:**

Vascular tortuosity.

**Results:**

Cumulative tortuosity index was the best metric produced by iROP-Assist for discriminating between OIR mice and NOX controls. Increased vascular tortuosity correlated with disease activity in OIR. Treatment of OIR mice with aflibercept rescued vascular tortuosity.

**Conclusions:**

Vascular tortuosity is a quantifiable feature of the OIR model that correlates with disease severity and may be quickly and accurately quantified using the iROP-Assist algorithm.

**Financial Disclosure(s):**

Proprietary or commercial disclosure may be found in the Footnotes and Disclosures at the end of this article.

Ischemic retinopathies such as diabetic retinopathy, retinopathy of prematurity (ROP), and retinal vein occlusions remain leading causes of blindness in the world.^1^^,^^2^ With > 1000 publications on the model since 1994, the murine oxygen-induced retinopathy (OIR) model is one of the most widely-utilized animal models in preclinical studies aimed at developing novel therapeutic strategies for ischemic retinopathies.^3^ Oxygen-induced retinopathy mice recapitulate key features of ischemic retinopathy— initial vaso-obliteration (VO) followed by neovascularization (NV)—and additional sequelae such as vascular leakage and proliferation.^4^^,^^5^ Ischemic retinopathies like ROP are characterized by aberrant NV and avascular zones, however, these conditions also present with increased vascular dilation and tortuosity. Tortuosity is a crucial indicator of disease severity, need to treat, and treatment response in ROP.^6^ Preclinical research in OIR mice typically report the effect of therapeutic interventions on rescuing NV and VO, but measurements of vascular tortuosity in OIR mice are less commonly reported.^7^, ^8^, ^9^, ^10^, ^11^, ^12^ In fact, a standardized methodology to calculate vascular tortuosity in OIR mice does not currently exist.

The current proof-of-concept study applied a previously published semi-automated computer-based image analysis approach toward quantifying vascular tortuosity in the murine retina. The correlation between retinal vascular tortuosity and disease activity in OIR mice was investigated using this novel quantification tool. Vascular studies using deep convolutional neural networks to quantify tortuosity in human retinal images of ROP can now outperform human experts.^13^ Using the outputs of this deep learning model, recent studies have been able to develop a quantitative metric of vascular tortuosity using an algorithm called iROP-Assist that takes into account features like vascular dilation and tortuosity.^14^ Such a metric does not exist to quantify tortuosity in OIR mice, the classic model for ROP. In the current study, we utilized the capabilities of iROP-Assist to generate a cumulative tortuosity index (CTI) measurement that may be used as a novel and standardized outcome measurement of vascular tortuosity in future experiments using the OIR model. Manually segmented OIR images were analyzed with the iROP-Assist algorithm to establish which measurements of tortuosity had the best discrimination between OIR mice and wild-type controls. The degree of retinal vascular tortuosity in OIR mice trended with the various stages of the model’s pathophysiology. Agents known to rescue NV in the OIR phenotype (aflibercept) were found to rescue CTI in OIR mice. Altogether, this work provides a new tool to quantify vascular tortuosity in OIR mice.

## Methods

### Animals

Age-matched C57BL/6 mice (The Jackson Laboratory, JAX) were subjected to normoxic (NOX) conditions or to the OIR model as previously described.^3^^,^^5^ In brief, pups were exposed to an atmosphere containing 75% oxygen from postnatal day (P) 7 to P12, after which they were returned to room air until euthanized at prespecified time points: P12 (immediately), P17, and P25. Using previously published methods,^5^ retinas were dissected from enucleated eyes by using fine brushes to separate and clean retina from choroid and sclera. Retinas were fixed in 4% paraformaldehyde on ice for 1 hour prior to overnight incubation in phosphate-buffered saline with Ca^2+^Mg^2+^ with 10 μg *Griffonia simplicifolia*-Isolectin B4 (I21412, Thermo Fisher Scientific). After being cut into 4 leaflets, retinas were flat-mounted for imaging using a Zeiss 710 confocal laser–scanning microscope with ZEN 2010 software (Zeiss) at 10x magnification and tile scanning (6 × 6 tiles).

Four groups of experiments were performed. In the first experiment, manual segmentation was performed by 4 graders on 10 NOX mice retinas and 10 OIR mouse retinas randomly selected from a previous dataset.^5^ Intergrader crossvalidation of the manual segmentation technique was achieved using the Dice coefficient. For the second experiment, 50 retinas from 50 individual NOX or OIR mice were randomly selected from P17 images in the same dataset. For the third experiment, NOX and OIR images were selected at P12, P17, and P25 from the same dataset. For timepoints at which the previously published dataset did not contain sufficient numbers of retinal images, additional retinas were prepared by the same authors using the same methodology. The last experiment used another previously published dataset of flat-mounted retinal images from 2 groups of 18 OIR mice intravitreally injected with aflibercept versus Immunoglobulin G contralaterally as control.^15^

### Manual Segmentation and Crossvalidation

Flat-mount images of NOX and OIR retinas were manually segmented by 4 authors (J.S.C., H.K.R.H., J.M., and S.P.) for large vessels using Adobe Photoshop (Adobe). In summary, large vessels were defined as those that originated from the disc center as well as large vessel branches. Capillaries, neovascular tufts, and areas of VO were not segmented. To ensure segmentation methodology was similar between graders, cross-validation was performed on a set of 10 images from each group that were segmented by all authors. Combinations of all vessel segmentations for each image were first manually reviewed for similarity by 2 independent graders (J.S.C. and J.M.). Vascular segmentation was then compared using Dice coefficient, which calculates the area of total overlap between the pixels segmented in 2 different images.

### Computer-based Analysis of Vascular Tortuosity

A previously published algorithm for calculating plus disease on fundus images of infants with ROP was adapted to quantify vascular tortuosity of all segmented images and the associated coordinates of the optic disk center.^14^ In summary, all unique pixels representing vessels from each segmentation were extracted to generate a graph of vessel segments. Point-based and vessel-based features such as integrated curvature (IC), CTI, and overall curvature (OC) were calculated from these vessel graphs using methods previously described by Ataer-Cansizoglu et al^16^ and definitions as described by Boton-Canedo et al^17^ and Han.^18^ Cumulative tortuosity index measures the mean cumulative sums of angles between segmented vessels normalized by vessel length, OC measures the mean angle curvature for all segments, and the IC is the integral of the OC.

To investigate the validity of vascular tortuosity as a quantifiable feature of OIR, an initial pilot study using 20 randomly selected images (10 NOX and 10 OIR) of a previously published dataset was conducted.^5^ The mean IC, CTI, and OC were calculated for all 20 images and compared between groups. After analyzing the data, CTI was selected as the test metric of choice for all subsequent experiments quantifying vascular tortuosity since its methodology is the most appropriate for calculation of vascular tortuosity.

### Quantification of NV and VO

Vaso-obliteration and NV ratios were calculated as previously described.^19^ In summary, a fully automated deep learning algorithm was used on images of retinal flat-mounts to generate segmentations for areas of NV and VO. The percentage of the images’ segmented area representing NV and VO were calculated as NV and VO ratios. These analyses were performed using a website hosting the deep learning system at http://oirseg.org/list.html.^19^ All images for both experiments were input into this algorithm for generation of NV and VO ratios, and manually reviewed by a 2 graders (J.S.C. and J.M.) to ensure that the segmentation accurately reflected pathology in the image. Of note, several NOX images were quantified by the deep learning algorithm to have high VO (likely due to areas of lighter pigmentation), but expert reviewers noticed that these images contained no VO as would be consistent with the natural physiology of vascular growth in NOX mice. For these images, the VO was adjusted to agree with the experience of expert graders.

### Statistics

All statistical analyses were performed using R 4.0.5 (R Foundation). For the pilot study and both experiments, mean CTIs, NV ratios, and VO ratios and their associated standard deviations (SDs) were calculated at each time point for the first experiment (P12, P17, and P25) and were stratified by NOX versus OIR in both experiments. Statistical significance for experiments containing 2 groups was assessed using an unpaired Student *t* test with statistical significance threshold of *P* < 0.05. One-way analysis of variance was used for comparison of ≥ 3 groups. Statistical significance was determined as ∗*P* < 0.05, ∗∗*P* < 0.01, ∗∗∗*P* < 0.001, and ∗∗∗∗*P* < 0.0001 in all figures.

### Study Approval

All experiments using animals were approved by Scripps Research Animal Care and Use Committee and the University of California at San Diego. Experiments were performed on C57BL/6 mice in accordance with the National Institutes of Health Guide for the Care and Use of Laboratory Animals (National Academies Press, 2011). Protocols were approved by the institutional review board at Scripps Research and Scripps Memorial Hospital, La Jolla, California, United States, and the University of California at San Diego. Study approval for previously published datasets was obtained as previously described.^5^^,^^15^

## Results

### Crossvalidation of Manual Segmentations

**Figure 1:**
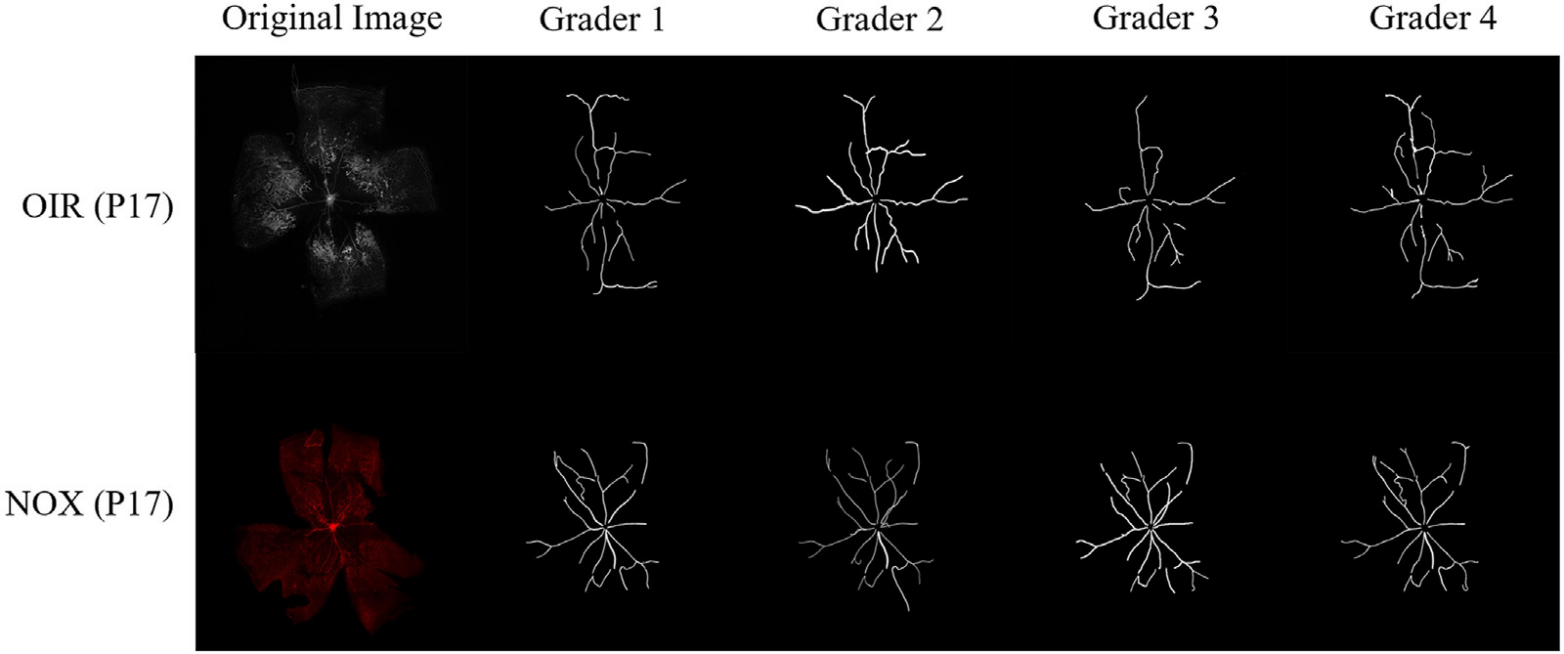
Representative segmentations of flat-mounted retinas of normoxic (NOX) and oxygen-induced retinopathy (OIR) mice from 4 independent graders. Oxygen-induced retinopathy (top) and NOX (bottom) mice were stained with GS-IB4 and flat-mounted for manual quantification by 4 independent graders. All segmentations were reviewed by an expert ophthalmologist. Following expert review of image segmentation from an ophthalmologist (J.C.), Dice coefficients were calculated to compare the similarity of vessel segmentations between all graders. GS-IB4 = *Griffonia simplicifolia*-Isolectin B4; P = postnatal day.

To ensure that manual segmentation of tortuosity was reliably quantified between graders, a cross-validation experiment was performed on a sample of randomly selected age-matched retinas of mice sacrificed at P12, P17, and P25 under NOX and OIR conditions from a previously published dataset.^5^ Manual vessel segmentation was performed by 4 graders (J.S.C., H.K.B., S.P., and J.M.). Crossvalidation of manual segmentations was achieved by comparing 20 flat-mounted retinas (10 NOX and 10 OIR) from the age-matched experimental dataset. All vessel segmentations were reviewed by 2 independent graders (J.S.C. and J.M.) to verify that selection of large vessels for segmentation was similar among all graders. For each image, all vessel segmentations produced by each grader were compared quantitatively using the Dice coefficient. Comparison of the Dice coefficient for all 6 combinations of 4 graders (0.79 ± 0. 13, 0.58 ± 0.27, 0.63 ± 0. 25, 0.61 ± 0.1, 0.61 ± 0.21, and 0.57 ± 0.24) demonstrated no significant differences in vascular segmentation (*P* = 0.24), suggesting manual segmentation was a reliable method for future experiments. Representative images of flat-mounted retinas and corresponding segmentations among all 4 graders are shown in Figure 1.

### Pilot Assessment of Vascular Tortuosity Metrics

**Figure 2:**
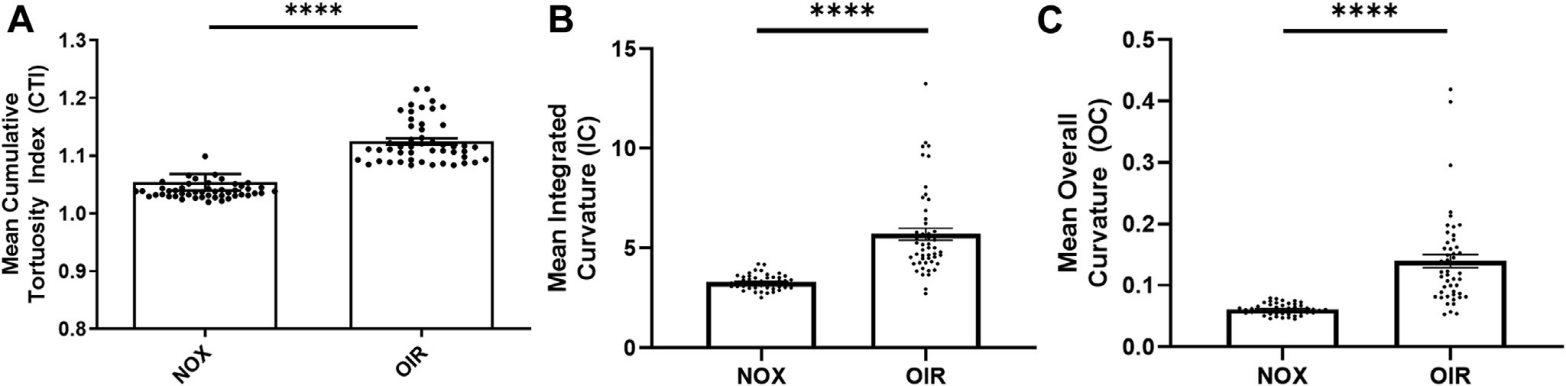
Quantification of vascular tortuosity in normoxic (NOX) and oxygen-induced retinopathy (OIR) mice using previously defined metrics. Manual segmentation of 50 NOX images and 50 OIR retinal images from mice sacrificed at postnatal day 17 in a previously published dataset^5^ were quantified using a published algorithm that extracts various tortuosity metrics including (**A**) cumulative tortuosity index, (**B**) integrated curvature, and (**C**) overall curvature. Two-tailed Student *t* test demonstrated statistically significant differences between all vascular tortuosity metrics when comparing images from NOX and OIR mice. Results were compared using a 2-tailed Student *t* test; error bars represent standard error of the mean; ∗∗∗∗*P* < 0.0001.

A pilot study was performed to investigate metrics of tortuosity including the IC, OC, and CTI on NOX versus OIR retinas from the same dataset.^5^ These metrics were derived from a previously published artificial intelligence algorithm that calculates various metrics to assess plus disease in ROP.^14^ As described by Boton-Canedo et al^17^ and Han,^18^ CTI measures the mean cumulative sums of angles between segmented vessels normalized by vessel length, OC measures the mean angle curvature for all segments, and the IC is the integral of the OC. All vascular tortuosity metrics demonstrated statistically significant differences (*P* < 0.0001) when discriminating vascular tortuosity between NOX and OIR mice (Fig 2). Cumulative tortuosity index was selected for use in all subsequent experiments since its methodology is the most intuitive for measuring vascular tortuosity.

### Assessment of Vascular Tortuosity Between Age-matched NOX vs. OIR Images

To investigate the validity of quantifying vascular tortuosity as an outcome measurement of OIR mice, manually segmented flat-mount images from age-matched NOX and OIR mice were analyzed at timepoints of pathophysiological relevance in the model (Table 1). Mice placed in hyperbaric oxygen on P7 were sacrificed on P12 immediately upon return to room air at the onset of the ischemic drive, on P17 at the peak of the neovascular phase, and on P25 when the retinal vasculature architecture is partially restored. These retinal images were obtained from a previous dataset^5^ with additional numbers obtained using the same methodology and by the same authors.

**Table 1:** Number of Normoxic and OIR Mice Used in Experiments Characterizing Neovascularization, Vaso-obliteration, and Cumulative Tortuosity Index

Age	Number of Retinas (n)	
	Normoxic Mice	OIR Mice
P12	42	34
P17	37	50
P25	48	32

OIR = oxygen-induced retinopathy; P = postnatal day.

**Figure 3:**
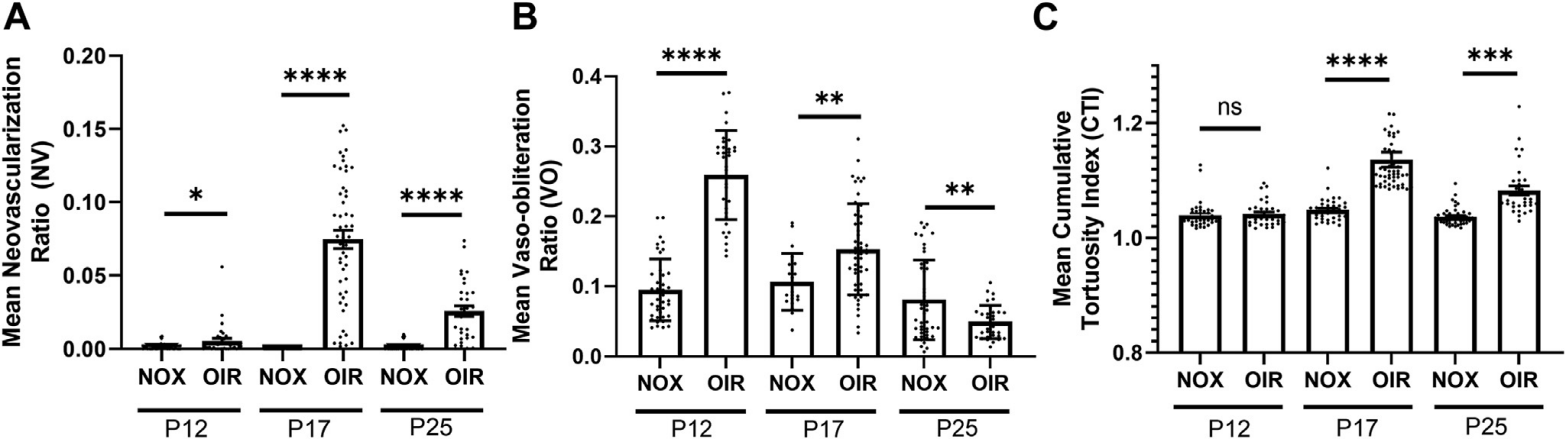
Characterization of neovascularization (NV), vaso-obliteration (VO), and vascular tortuosity in age-matched normoxic (NOX) and oxygen-induced retinopathy (OIR) retinas at postnatal day (P) 12, P17, and P25. **A**–**C,** Neovascularization, VO, and vascular tortuosity measured as cumulative tortuosity index (CTI) demonstrated changes congruent with the pathophysiology of OIR. **A, B,** Quantified using a publicly available deep learning algorithm, the (**A**) NV and (**B**) VO of NOX and OIR mice were consistent with previous reports.^5^ In OIR mice, NV peaks at P17 and is partially resolved by P25 while VO peaks at P12 and subsequently decreased over time. **C,** Quantification of CTI using a previously published algorithm demonstrated that similar to NV, vascular tortuosity is increased at P17 and partially resolves by P25.^14^ The images from which this data was collected were used in a previously published manuscript^5^ with additional mice prepared using the same methodology and by the same authors. P12: n = 42 NOX retinas, n = 34 OIR retinas; P17: n = 37 NOX retinas, n = 50 OIR retinas; P25: n = 48 NOX retinas, n = 32 OIR retinas. Results were compared using a 2-tailed Student *t* test; error bars represent standard error of the mean; ns = non-significant, ∗*P* < 0.05, ∗∗*P* < 0.01, ∗∗∗*P* < 0.001, ∗∗∗∗*P* < 0.0001.

The mean ± SD NV ratio for NOX images at all timepoints was 0, and the mean ± SD NV ratio for OIR images was 0.01 ± 0.01 at P12 (*P* = 0.01), 0.08 ± 0.04 at P17 (*P* < 0.0001), and 0.03 ± 0.02 at P25 (*P* < 0.0001) (Fig 3A). The mean ± SD VO ratio for NOX and OIR images was 0.09 ± 0.04 versus 0.26 ± 0.06 at P12 (*P* < 0.0001), 0.11 ± 0.04 versus 0.15 ± 0.07 at P17 (*P* = 0.004), and 0.08 ± 0.06 versus 0.05 ± 0.02 at P25 (*P* = 0.004) (Fig 3B). The mean ± SD CTI for NOX and OIR images was 1.04 ± 0.02 versus 1.04 ± 0.02 (*P* = 0.6) at P12, 1.05 ± 0.01 versus 1.14 ± 0.09 (*P* < 0.0001) at P17, and 1.04 ± 0.01 versus 1.08 ± 0.04, respectively (*P* < 0.0001), at P25 (Fig 3C). Together, these data suggest that vascular tortuosity is a valid and distinguishable feature of the OIR model at P17 and P25, time points of pathologic significance. Analogously to NV in the OIR model, vascular tortuosity increases from P12 to P17 and then regresses.

### Vascular Tortuosity in Mouse Eyes Treated With Aflibercept vs. Immunoglobulin G Control

The next series of experiments were performed to investigate the effects of known therapeutic agents on vascular tortuosity in OIR mice using another previously published dataset.^15^ Previous reports have demonstrated that intravitreal injections of aflibercept decrease NV and retinal inflammation in OIR mice, but the effects of aflibercept on vascular tortuosity remain unknown.

**Figure 4:**
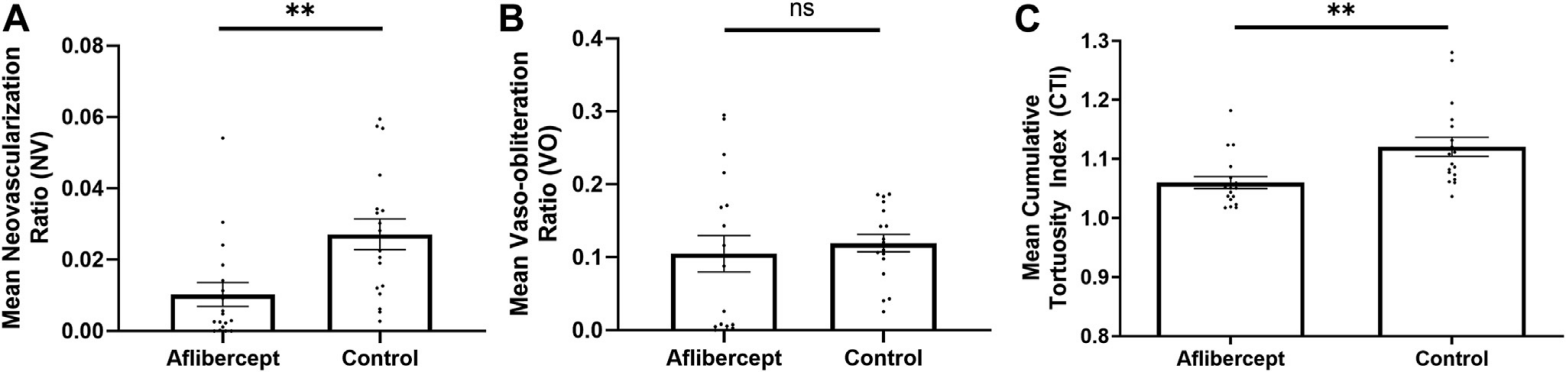
Characterization of neovascularization (NV), vaso-obliteration (VO), and vascular tortuosity in oxygen-induced retinopathy mice following treatment with aflibercept. **A**–**C,** Intravitreal injections of aflibercept improved retinal NV and cumulative tortuosity index (CTI) while the effect on VO did not reach statistical significance. A total of 18 images of eyes treated with aflibercept and 18 images of eyes treated with Immunoglobulin G control were manually segmented. These images were part of the authors’ previously published dataset.^15^ (**A**) Neovascularization, (**B**) VO, and (**C**) CTI were calculated for all images and compared using a 2-tailed Student *t* test; error bars represent standard error of the mean; ns = non-significant. ∗*P* < 0.05, ∗∗*P* < 0.01, ∗∗∗*P* < 0.001, ∗∗∗∗*P* < 0.0001.

Eyes of OIR mice placed in hyperbaric oxygen on P7 were intravitreally injected with aflibercept with contralateral eyes injected with Immunoglobulin G as control on P12 (immediately upon return to room air) and sacrificed on P17 for preparation of retinal flat-mounts. A total of 36 images, 18 eyes treated with aflibercept and 18 treated with Immunoglobulin G control, were manually segmented and CTI was calculated using iROP-Assist. Neovascularization and VO ratios were calculated using previously published methods.^19^ The mean ± SD NV ratio for aflibercept versus control was 0.01 ± 0.01 versus 0.03 ± 0.02 (*P* = 0.003) (Fig 4A). The mean ± SD VO ratio for aflibercept versus control was 0.10 ± 0.14 versus 0.12 ± 0.05 (*P* = 0.6) (Fig 4B). Eyes treated with aflibercept demonstrated a mean ± SD CTI of 1.05 ± 0.04, whereas the control eyes demonstrated a mean CTI of 0.12 ± 0.06 (*P* = 0.002) (Fig 4C). Overall, these data suggest that treatment with anti-VEGF agents attenuate vascular tortuosity of the OIR model. This effect is analogous to the effect of anti-VEGF on vascular tortuosity in ROP.^20^ Reduction in vascular tortuosity may serve as a useful quantitative outcome parameter in future experiments testing therapeutics in OIR mice.

## Discussion

For years, the lack of a standardized definition of vascular tortuosity conditioned clinical specialists with subjective and thus varied conceptualizations of what constitutes tortuosity, leading to high inter- and even intraexpert variability in its quantification.^21^^,^^22^ Such ambiguity limited use of this metric as both a research and clinical tool. With the advent of explainable deep learning methodologies such as iROP-Assist, computational approaches capable of quantifying vascular tortuosity in humans have become clinically relevant.^6^ In addition to its emerging role in the management of ROP, retinal vascular tortuosity is a clinical biomarker for numerous other vascular and systemic diseases including diabetic retinopathy, cerebrovascular disease, stroke, kidney dysfunction, and ischemic heart disease.^21^^,^^23^, ^24^, ^25^, ^26^ The importance of vascular tortuosity to clinical medicine is an exciting and ongoing development. At the same time, the adaptation of these tools for use in preclinical studies on animal models of disease allows translational research to draw upon lessons learned from the clinics. The current work, therefore, aimed to bring findings from the bedside back to the bench by adapting the iROP-Assist system to quantify vascular tortuosity in the retina of OIR and NOX mice. These data suggest that vascular tortuosity in OIR mice can be quantified using computer-based imaging analysis.

The majority of literature using OIR mice report the retinal area covered by NV or VO on P17, typically in order to investigate phenotypic rescue following administration of a therapeutic agent on P12. Only a few studies have considered vascular tortuosity as an outcome measurement for the OIR model, and these publications typically report vascular tortuosity either by using an operationally defined scoring system or by manually calculating the ratio of tortuous to straight major vessels leaving the optic disc on a small selection of vasculature.^7^, ^8^, ^9^, ^10^, ^11^, ^12^ Prior work using machine learning to quantify vascular tortuosity did not publish their algorithm, limiting the reproducibility of results.^27^ In contrast, the vascular tortuosity algorithm from iROP-Assist analyzes all retinal vasculature, is publicly available, and has been validated on ∼100 fundus images in human ROP. The current study provides a quantitative CTI by using a computer-based image analysis algorithm that employs a feature-extraction-based approach to take into account all the major superficial vasculature within a retinal image. Coupled with our recent publication automating the generation of vessel segmentation,^28^ this tool can provide a rapid, standardized, and accurate measurement of vascular tortuosity.

Similar to NV, the CTI of OIR mice increases after removal from hyperoxia on P12, is elevated at P17, and spontaneously improves, as demonstrated by decreased CTI at P25. Intravitreal injection of the anti-VEGF agent aflibercept rescued both the NV as well as the CTI of OIR mice at P17. There are multiple possible explanations regarding the effects of anti-VEGF treatment on vascular tortuosity. Anti-VEGF therapeutic agents may directly attenuate the development of vascular tortuosity. Alternatively, vessel tortuosity may be dependent on retinal NV and regression of NV may reduce tortuosity.

This work applied techniques developed to quantify tortuosity in human ROP to its preclinical disease model of OIR in mice. Oxygen-induced retinopathy mice developed tortuosity that correlated temporally with NV and treatment of the model with aflibercept rescued both outcome metrics. Given the clinical importance of assessing tortuosity in human ROP, future work using this tool to characterize CTI in OIR mice may present a new and promising quantitative metric used in preclinical studies evaluating the efficacy of novel therapeutics in ROP.
